# Students’ Trait Emotional Intelligence and Perceived Teacher Emotional Support in Preventing Burnout: The Moderating Role of Academic Anxiety

**DOI:** 10.3390/ijerph17134771

**Published:** 2020-07-02

**Authors:** Luciano Romano, Xin Tang, Lauri Hietajärvi, Katariina Salmela-Aro, Caterina Fiorilli

**Affiliations:** 1 Department of Human Sciences, University of Rome LUMSA, 00193 Rome, Italy; fiorilli@lumsa.it; 2 Faculty of Educational Sciences, University of Helsinki, 10014 Helsinki, Finland; xin.tang@helsinki.fi (X.T.); lauri.hietajarvi@helsinki.fi (L.H.); katariina.salmela-aro@helsinki.fi (K.S.-A.)

**Keywords:** school burnout, trait emotional intelligence, teacher emotional support, academic anxiety, high school students

## Abstract

The current study sought to investigate the role of trait emotional intelligence and perceived teacher emotional support in school burnout. Furthermore, the moderating role of academic anxiety in these relationships was examined. A sample of 493 Italian high school students (81.9% female) aged 14–19 years (M = 16.27, SD = 1.48) was involved in the study. A latent moderated structural equation approach was performed to test the hypothesized model. The results showed that both trait emotional intelligence and perceived teacher emotional support were negatively associated with school burnout. Moreover, academic anxiety moderated the relation between perceived teacher emotional support and school burnout. Specifically, when the level of anxiety was high, the protective role of perceived teacher emotional support toward burnout was weakened. Findings are discussed in light of the protective role of resources on burnout and considering the detrimental impact of academic anxiety in school settings.

## 1. Introduction

Over-exposure to academic stress can result in school burnout—a state of physical, mental, and emotional exhaustion [1,2]. With this regard, authors have focused on the factors and underlying processes involved in this syndrome, showing the pivotal role of individual and external (perceived) resources in its prevention [3,4]. Specifically, the trait emotional intelligence, as an individual/personal resource and a constellation of emotion-related dispositions, appears to shape the perception and interpretation of stressful events as well as helping to deal with them [5]. Moreover, previous studies have shown that students who perceived their teachers as emotionally supportive may feel more shielded than their peers who perceive their teacher as less supportive, and are more capable of handling academic requirements [6]. Besides the protective role of resources, according to the demands-resources model [4], it is important for the direct and synergistic effect of demands to be examined simultaneously. Thus, in this study the role of academic anxiety on school burnout was examined given the detrimental effect of excessive academic anxiety on students’ daily life [7].

### 1.1. School Burnout and Students Resources

According to the existing literature and in light of the demands-resources model applied to the educational context [4], school burnout could be depicted as a syndrome that depends on the imbalance between external demands and available resources, both school-related and personal ones [4,8]. When exposed to high levels of chronic stress, students start to feel physically and mentally overwhelmed and exhausted, losing all their capability to deal with academic demands. Furthermore, burned-out students experience a sense of cynicism and meaningless toward the school, which acts as a last, wrong attempt to counteract and cope with the experienced emotional exhaustion. Finally, they feel useless and inadequate as a student, losing satisfaction and personal accomplishment [2,9] ([10] for a review). Several studies have shown the detrimental effects of burnout in adolescents in terms of dropout, poor performance, and other clinical or subclinical conditions later on [11,12,13,14,15]. According to Alarcon and colleagues [3], who applied the conservation of resources theory [16] to educational tracks, school burnout can be considered as the product of the consumption of available resources. Thus, the capability to react to academic strain and to avoid burnout depends on the amount of individual and external resources a student can count on.

Among the individual resources, previous studies have highlighted the role of trait emotional intelligence when dealing with emotionally taxing situations in school settings (e.g., [17]). Research usually conceptualizes emotional intelligence in two different ways due to separate theoretical frameworks, historically at odds with each other. In essence, the first one, related to the studies of Mayer and Salovey [18], considers emotional intelligence as a cognitive ability, thus measured by performance-based tests to capture “maximal” performance. The second one, related to the conceptual framework of Petrides [19], considers emotional intelligence as a personality trait located at the lower levels of personality hierarchies, therefore measured by self-report questionnaires to capture “typical” performance [20,21]. According to Petrides [19], the trait emotional intelligence is regarded as self-efficacy in processing emotion-laden information [19,22]. Mikolajczak [23], with the three-level model of emotional intelligence, has tried to solve the debate between the two theories, positing that the different types of emotional intelligence should coexist in a unified framework. Thus, according to the author, when dealing with emotions, knowledge (what I know), abilities (what I can do), and dispositions (what I do) have to be considered as parts of a unique process [23]. Despite this, later studies have shown that the two constructs do not strongly correlate with each other and that their different associations with the same variables could be reflective of different processes [24,25,26]. A recent literature review by Petrides and colleagues [27] has deepened the relationship between trait emotional intelligence and adjustment in school contexts. In detail, students with high trait emotional intelligence report higher prosocial behaviors towards teachers and peers, lower rates of unauthorized absences, and have been less expelled from school than their low emotionally intelligent peers, thus reflecting a higher school adaptation [28,29,30]. Moreover, further studies have demonstrated that trait emotional intelligence is negatively related to perceived stress and depressive symptoms in students [31,32]. According to Saklofske and colleagues [33], a high trait emotional intelligence in students facilitates the use of the effective emotion regulation strategies to cope with school-related stressors. Specifically, recent studies have shown that, when dealing with undue school pressures, students with high trait emotional intelligence are more inclined to avoid potentially damaging emotion-focused strategies (i.e., rumination), and this, in turn, facilitates the achievement of their academic goals [34]. High trait emotional intelligent students, indeed, compared to their counterparts, seem to be more able to evaluate demanding situations as a challenge rather than a threat, therefore being more shielded against chronic stress [20,35,36]. Moreover, a recent study has highlighted that high school students with high trait emotional intelligence levels also report low levels of school burnout [37].

As to external resources, previous studies have highlighted that perceived teacher emotional support is related to a plethora of positive school outcomes [15,38,39,40,41,42]. For instance, scholars have demonstrated a correspondence between teachers who show autonomy, supporting, and caring instructional behaviors and students’ perceptions of autonomy and support, with significant implications for students’ engagement and performance (e.g., [43]). Teacher emotional support usually refers to students’ perception of their teacher as warm, friendly, and caring [44,45,46]. According to Pianta and Hamre [39], teacher emotional support consists of three dimensions: The first one, positive climate, refers to the ability of teachers to create positive interactions with their students. Besides this, teacher sensitivity concerns the extent to which a teacher is prone to respond to students’ academic and emotional needs. Finally, a regard for adolescent perspective pertains to the degree to which teachers promote the autonomy and general development of their students [46,47,48,49]. Previous studies have shown that, compared to other sources of support, high school students’ perception of teacher emotional support is more strongly associated with low levels of emotional exhaustion and high subjective well-being (e.g., [50,51]). Moreover, further scholars have shown that a high perceived teacher emotional support buffers the negative impact of stressful life events on depressive symptoms [52]. Students who employ emotional support seeking strategies are more likely to be shielded against excessive academic strain [53]. Further studies have shown that students who benefit from caring teachers could broaden their personal resources, thoughts, and behaviors, thus gaining a better adaptation to the school setting and taxing academic demands [54,55]. Specifically, students who positively perceived the emotional support from their teachers are more protected against maladjustment and school burnout than their counterparts [56,57].

### 1.2. The Moderating Role of Academic Anxiety

The existing literature considers academic anxiety as a general feeling of being nervous and worried in the academic context due to external demands, such as tests and assignments as well as the high pressure in obtaining excellent grades (e.g., [58,59]). It has been widely demonstrated in the academic literature (e.g., [60,61,62]) that small amounts of academic anxiety could promote better achievement, fostering students’ attention and concentration. Despite this, further studies have shown that excessive academic anxiety levels are related to various adverse outcomes in the school context [63,64,65]. Effectively, students who experience negative emotions and worries related to their school commitments are more prone to experience burnout than their counterparts [35,63,66].

In this regard, it is possible that, although typically resources act as protective factors against school burnout, students in the context of overwhelming demands can become so anxious that also their perceptions of resources, individual as well as external, might be weakened, therefore making them more exposed to the risk of burnout (e.g., [67]). Previous scholars have shown that individuals who experience high anxiety, as opposed to their counterparts, tend to have a biased perception of their emotions, present lower levels of emotional clarity and awareness, and are more likely to refuse the ongoing emotions they are experiencing [68]. Concerning the school setting, the existing literature underlines that, when feeling extremely anxious, some students are inclined to adopt adverse and ineffective emotional regulation strategies—i.e., suppression—therefore lessening their ability to deal effectively with academic stressors (e.g., [69,70,71]). Further studies, indeed, have highlighted that students with high anxiety levels also report low trait emotional intelligence and wrong emotion regulation strategies usage, thus being more at risk for school refusal and burnout [72,73].

Moreover, even though highly emotionally supported students are supposed to be more shielded against adverse academic outcomes (e.g., [74,75,76]), in high-anxiety conditions students could be less capable of benefiting from their teachers’ emotional support, thus being more vulnerable to school burnout. Recent studies, indeed, have highlighted that when extremely anxious, students show more reluctant and rejecting behavior toward school and instructions than their less anxious peers (e.g., [77]). Besides this, students with severe anxiety tend to avert interaction and exhibit low social and emotional competences (e.g., [78,79]). Effectively, students with high anxiety levels prefer avoidant rather than support seeking strategies (i.e., teacher support) [80,81].

### 1.3. Aims and Hypotheses

Based on the abovementioned literature review, the present study aimed to examine the relations between external and internal resources and burnout, taking into account anxiety as a possible moderator.

In detail, the following set of hypotheses were formulated:

Trait emotional intelligence and perceived teacher emotional support were both negatively related to school burnout. Conversely, academic anxiety was positively related to students’ burnout levels (Hypothesis 1).

Academic anxiety moderated the relationships between trait emotional intelligence and school burnout (Hypothesis 2). In detail, it was expected that high (vs. low) anxiety would lessen the effect of trait emotional intelligence on school burnout.

**Figure 1:**
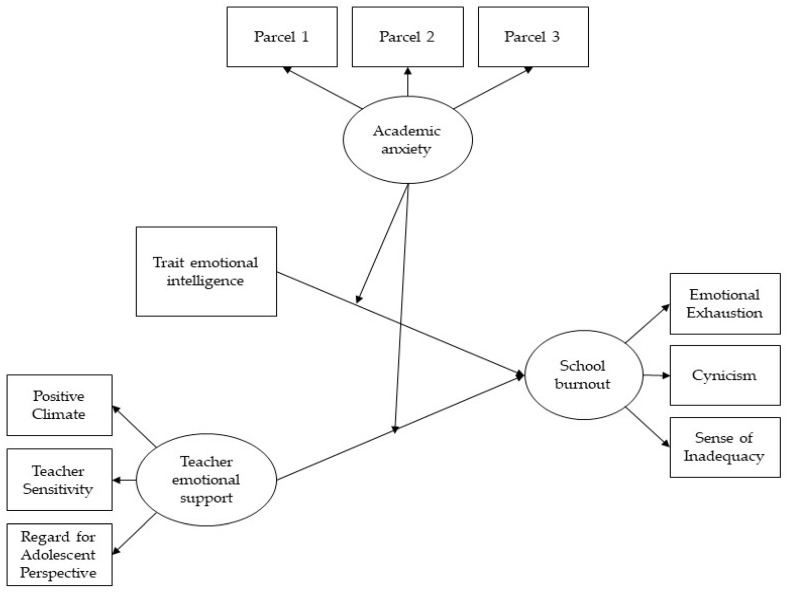
A conceptual model of the hypothesized moderating effect of academic anxiety on the links between trait emotional intelligence and perceived teacher emotional support with school burnout.

Academic anxiety moderated the relationships between perceived teacher emotional support and school burnout (Hypothesis 3). In detail, it was expected that high (vs. low) anxiety students would perceive less emotional support from their teachers, thus being more exposed to school burnout. In order to make the model more conservative, students’ age and gender were used as control variables. The hypothesized model is shown in Figure 1.

## 2. Materials and Methods

### 2.1. Participants and Procedure

This study used a cross-sectional descriptive design with a convenience sample. The participants that took part in the study were 493 Italian high school students (81.9% female) aged 14–19 years (M = 16.27, SD = 1.48). There were no missing data. Initially, the sample was composed of 497 participants, but, due to outliers, 4 participants were excluded from the analyses. These students belonged to two different high schools in Central (87.6%) and Southern Italy (12.4%). In detail, 69.8% of the participants attended a human sciences high school (78% female), while 30.2% attended a high school specializing in classics subjects (22% female). The research protocol received the approval of the school council as well as of the school principal. Only the students who complete informed consent took part in the study. Furthermore, only underage students who provided the informed consent signed by their parents could participate in the study. Anonymity and confidentiality standards were assured to all the subjects involved in the study. Students complete the administrations in their classrooms, with a paper-pencil approach and during school hours. The teachers were not allowed to stay in classrooms, and the students were assured that only the research team could have access to the collected data. The researcher provided all the necessary information to complete the study and was present during the administrations to provide further information in case of need. The study was conducted in compliance with the Declaration of Helsinki of 1964 and its latest version, and all the study procedures received approval from the Ethics Committee of Lumsa University of Rome, Italy.

### 2.2. Instruments

Trait emotional intelligence: Trait Emotional Intelligence Questionnaire Short Form (TeiQue-SF; [19]), in its Italian validated version [82], was used to assess trait emotional intelligence’s global score. TeiQue is a self-report questionnaire composed of 30 items on a 7-point Likert scale (1 = “I totally disagree”, 7 = “I totally agree”). An example of an item is: “I usually find it difficult to regulate my emotions”. The TeiQue-SF could provide the scores of the four trait emotional intelligence subdimensions (Emotionality, Sociability, Well-being, and Self-control) as well as a global trait emotional intelligence score. In previous studies, it has been widely used as a unidimensional global measure to assess trait emotional intelligence (for more detail, see [83]) and it has been recently used in the Italian context [37,84]. In the current study, Cronbach’s alpha was 0.82.

Teacher emotional support: Teacher emotional support was measured by the Teacher Emotional Support Scale used in Schenke et al. [49] in its Italian version [47]. The instrument is a 15-item self-report questionnaire based on a 5-point Likert scale (1 = “Not at all true”, 5 = “Very true”), and measuring students’ perceptions of the emotional support they receive from their teachers. It is composed of three subscales: 5 items for Positive Climate (“Our teachers treat everyone in this class fairly”), 6 items for Teacher Sensitivity (“Our teachers consider students’ feelings”), and 4 items for Regard for Adolescent Perspective (“Our teachers allow us to discuss our work with classmates”). In the validation study of the Italian version, the confirmatory factor analysis supported the three-dimensional structure of the construct, showing acceptable fit-indexes (CFI = 0.918, TLI = 0.901, RMSEA = 0.086, and SRMR = 0.063), as well as a good reliability (α= 0.91 for the total score, α= 0.74 for Positive Climate, α= 0.90 for Teacher Sensitivity, and α= 0.81 for Regard for Adolescent Perspective). In the present study, the fit-indices from the confirmatory factor analysis were the following: χ^2^ (88) = 388.463, *p* < 0.001; RMSEA = 0.08; SRMR = 0.07; CFI = 0.91; TLI = 0.89. Cronbach’s alpha was 0.93 for the total score, 0.82 for Positive Climate, 0.90 for Teacher Sensitivity, and 0.82 for Regard for Adolescent Perspective.

Academic anxiety: Academic anxiety was evaluated by the anxiety subscale of the Italian Questionnaire for Anxiety and Resilience (QAR; [85]). It consists of 7 items on a 5-point Likert scale (1 = “Not at all”, 5 = “Totally”). An example of an item is: “The closer the date of an exam/verification in class, the more I get anxious”. The scale has been previously used in the Italian context [66,86]. In the present study, the fit-indices from the confirmatory factor analysis were the following: χ^2^ (9) = 35.991, *p* < 0.001; RMSEA = 0.07; SRMR = 0.03; CFI = 0.98; TLI = 0.95. Cronbach’s alpha was 0.88.

School burnout: School burnout was measured by the School Burnout Inventory (SBI; [2]) in its Italian validated version [1]. This self-report questionnaire consists of 9 items on a 6-point Likert scale (1 = “I totally disagree”, 6 = “I totally agree”). The instrument evaluates three core aspects of school burnout: 4 items for Emotional Exhaustion (“I often sleep poorly because of all the problems related to the study ”), 3 items for Cynicism (“I feel a lack of motivation in my schoolwork and often think of giving up”), and 2 items for Sense of Inadequacy (“I used to have higher expectations of my schoolwork than I do now”). The SBI has been previously administered to Italian students [87]. In the present study, the fit-indices from the confirmatory factor analysis were the following: χ^2^ (25) = 105.089, *p* < 0.001; RMSEA = 0.08; SRMR = 0.04; CFI = 0.94; TLI = 0.91. Cronbach’s alpha was 0.85 for the total score, 0.76 for Emotional Exhaustion, 0.82 for Cynicism, and 0.65 for Sense of Inadequacy.

### 2.3. Analysis Plan

In order to verify the adequate normality of the studied variables, preliminary descriptive analyses, such as skewness, kurtosis, the Kolmogorov–Smirnov test [88], and the Shapiro–Wilk test [89], were performed using SPSS v. 21.0 (IBM, Armonk, NY, USA). Although the skewness and kurtosis were not >2, the Kolmogorov–Smirnov test and Shapiro–Wilk test revealed *p* < 0.05 for all the studied variables. Thus, assuming the non-normal distribution of the data, a robust maximum likelihood estimation (MLR, [90]) was used. Moreover, the Spearman correlation was performed to test the associations among the variables.

Regarding the model specification, both trait emotional intelligence and teacher emotional support were the predictors, academic anxiety was the moderator variable, and school burnout was the outcome. Age and gender were used as control variables in all the models. For trait emotional intelligence, a composite score was used to obtain a global score due to the scarce reliability of three of the four trait emotional intelligence subdimensions (α < 0.60 for Emotionality, Sociability, and Self-control) and in light of the considerations of Petrides [19]. In his study, the author asserted that, although the TeiQue-SF could be used to obtain the four factors, these tend to have considerably low reliability. As such, it has been used as a single composite score that usually presents excellent psychometric properties [19,91,92].

Teacher emotional support and school burnout were both modeled as latent variables, with the composite scores of their three subscales as indicators. Similarly, the items parceling procedure was followed for academic anxiety, and three parcels were used. The item aggregation into parcels was used in order to avoid model non-identification problems and for parsimony purposes. The items aggregated were randomly selected; the parcels had a similar accountability and contained a comparable number of items [93].

In order to verify the hypothesized model, a three-step procedure was followed using Mplus 8.3 [90]. First, the goodness-of-fit of the model without interaction (M0) was estimated using the following fit indices: a chi-square (*p*-value > 0.05 indicate a good fit), the comparative fit index (CFI) and the non-normed fit index (TLI) (values > 0.90 indicate a good fit; values > 0.95 indicate a very good fit), the root mean square error of approximation (RMSEA), and the standardized root mean square residual (SRMR) (values < 0.08 indicate a good fit, values < 0.05 indicate a very good fit).

Second, a latent moderated structural equation approach (LMS, [94]) was performed to estimate the main and interaction effects of academic anxiety on the relationship between the trait of emotional intelligence, teacher emotional support, and school burnout (M1).

Third, since LMS does not provide conventional fit indices, the log-likelihood (LL) difference test (Δ-2LL, [95]) was used to verify the improvement of the structural equation model with latent interaction (M1) compared to the structural equation model without the latent interaction term (M0).

Furthermore, when the interaction term was significative, the results were plotted in order to understand the relationship between the independent variable (IV) and the dependent variable (DV) at different levels of the moderation term (1 standard deviation above and below the mean). Finally, simple slope analyses were performed to verify the significance of the relationship between IV and DV at both of these two levels.

## 3. Results

### 3.1. Descriptives Statistics and Correlations

Table 1 reports the mean, standard deviations, skewness, kurtosis, and correlations. Gender variable was treated as a dummy variable, where 0 was the value attributed to females and 1 the value attributed to males.

Trait emotional intelligence was positively related to perceived teacher emotional support (*r* = 0.18, *p* < 0.001), while it was negatively related to academic anxiety (*r* = −0.398, *p* < 0.001) and school burnout (*r* = −0.40, *p* < 0.001). Moreover, the perceived teacher emotional support was negatively related to academic anxiety (*r* = −0.13, *p* < 0.01) and school burnout (*r* = −0.36, p < 0.001). Finally, academic anxiety was positively associated with school burnout (*r* = 0.47, *p* < 0.001). Age was negatively related to teacher emotional support (*r* = −0.11, *p* < 0.05) and positively related to school burnout (*r* = 0.24, *p* < 0.001). Furthermore, while trait emotional intelligence (*r* = 0.11, *p* < 0.05) and perceived teacher emotional support (*r* = 0.095, *p* < 0.05) were slightly high in males, academic anxiety (*r* = −0.25, *p* < 0.001) and school burnout (*r* = −0.17, *p* < 0.001) were strongly higher in female than male participants.

### 3.2. Structural Model for Testing Moderation Effects

The model without the latent interaction term (M0) showed a good adjusted model. In detail, χ^2^ (42) = 168.183 *p* < 0.001; root mean square error of approximation (RMSEA) = 0.07; standardized root mean square residual (SRMR) = 0.06; comparative fit index (CFI) = 0.94; non-normed fit index (TLI) = 0.91.

The results from the LMS are presented in Table 2.

**Figure 2:**
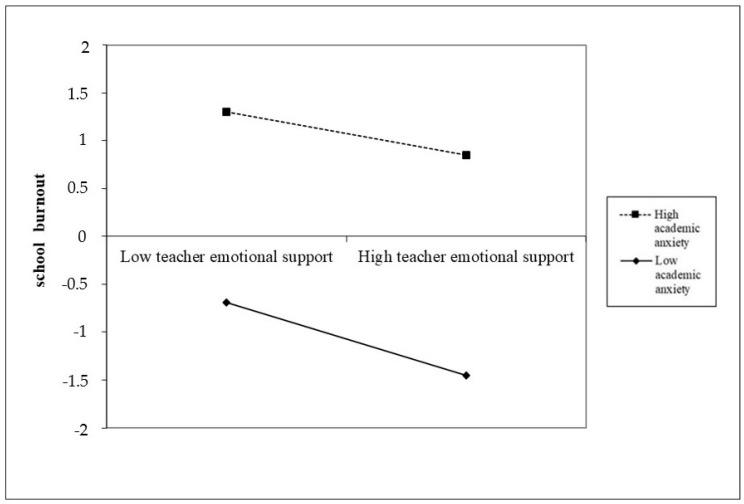
Latent interaction of teacher emotional support and academic anxiety on school burnout. Note. High academic anxiety = +1 standard deviation (SD); low academic anxiety levels = −1 standard deviation (SD).

Since the Δ-2LL test was significant (*p* = 0.002), it was possible to assume that the hypothesized interaction model (M1) represents an improvement to the data compared to the model without latent interaction terms (M0). In detail, trait emotional intelligence (*β* = −0.19, *p* < 0.001) and teacher emotional support (*β* = −0.29, *p* < 0.001) were both negatively related to school burnout, while academic anxiety was positively related to school burnout (*β* = 0.51, *p* < 0.05). Age but not gender positively predicted school burnout (*β* = 0.22, *p* < 0.001). Besides this, academic anxiety moderated the effect of teacher emotional support on school burnout (*β* = 0.13, *p* ≤ 0.01) (Figure 2). Furthermore, the simple slope analyses revealed that the inverse relation between teacher emotional support and school burnout was stronger at lower levels of academic anxiety (*β* = −0.38, *p* < 0.001) than at higher levels of academic anxiety (*β* = −0.22, *p* < 0.001). Finally, the results indicate that there was no statistically significant moderation of academic anxiety in the relationship between trait emotional intelligence and school burnout (*p* > 0.05).

## 4. Discussion

The present study explored the relationships among individual resources (i.e., trait emotional intelligence), external resources (i.e., perceived teacher emotional support), academic anxiety, and school burnout in a sample of high school students. Furthermore, as the main focus of the current study, the moderating role of academic anxiety on both individual and external resources towards school burnout was investigated. The effects of age and gender were controlled for in the model. Overall, the findings support the correlational hypotheses and partially confirmed the moderating hypotheses.

Our results supported Hypothesis 1. Students with higher trait emotional intelligence were more shielded against school burnout than their counterparts. Consistent with previous findings, indeed, students with high emotional self-efficacy, by using the correct emotional strategies, are more likely to handle the emotional burden related to school demands and are less inclined to feel overwhelmed (e.g., [27,96]). Furthermore, perceived teacher emotional support was negatively related to school burnout. Previous scholars, indeed, have highlighted that students’ perception of teachers’ emotional support was inversely related to emotional problems and adverse school outcomes (e.g., [97]). Moreover, students who perceive their teacher as caring and attentive to their emotional needs may feel more shielded and have further possibilities to counteract the strain and pressure of excessive school requirements (e.g., [47,98,99,100]). Besides this, considering that emotional consumption is the first and foremost component experienced by burned-out students [101], the ones who perceived high emotional and caring behavior from their teachers are more likely to broaden their emotional resources and overcome the burnout-related exhaustion (e.g., [3]).

Consistent with previous findings (e.g., [102,103]), academic anxiety was positively associated with school burnout. Highly anxious students in our sample, indeed, were also more likely to show high school burnout. Students with high anxiety levels experience excessive worries about school tasks and are more inclined to use the wrong strategies to react to harmful feelings [104,105]. Therefore, they may overestimate the negative emotions related to school demands and underestimate their ability to deal with them, thus being more exposed to burnout.

The main findings of this study concern the moderating role of academic anxiety. Contrary to our expectations, Hypothesis 2 was not supported, and academic anxiety did not affect the relationship between trait emotional intelligence and school burnout. Thus, the protective role of trait emotional intelligence on school burnout was not moderated by students’ levels of academic anxiety. According to some authors [106], due to the dispositional and trait-based facets of peoples’ trait emotional intelligence, it may persist over and beyond concurrent emotions, such as anxiety, when dealing with taxing conditions. In other words, highly emotionally intelligent students are largely already without anxiety, thus making us difficult to find the interaction between emotional intelligence and academic anxiety. Similarly, previous scholars have shown that, when controlling for personality measures, the association between trait emotional intelligence and anxiety was substantially reduced, thus suggesting that more comprehensive personality measures could intercept variance in anxiety better than emotional intelligence [107,108].

Conversely, and supporting Hypothesis 3, academic anxiety moderated the impact of external resources, such as perceived teacher emotional support, on school burnout, therefore lessening their protective role. Similar results were found in clinical samples, showing that extremely anxious individuals failed in perceiving social support and were thereby highly vulnerable to stress [109]. As mentioned before (e.g., [81]), highly anxious students are more likely to use avoidant strategies compared to their non-anxious peers. Further studies have shown that anxious and avoidant students report lower levels of global support (e.g., [110]), and seem to be less able to perceive and take advantage of the emotional support received than non-anxious and non-avoidant students [111]. Moreover, in the face of threatening events this kind of student tends to perceive others as less supportive [112]. Therefore, the more students are suffering from extreme anxiety, the less they perceive teachers as emotionally supportive, which in turn leads to high burnout levels.

The present finding sheds light on the pervasive role of anxiety in students’ daily life, showing how it could deplete the perception of resources coming from the school context and lead quickly to burnout. Thus, future interventions focused on students’ burnout prevention through the empowerment of teachers’ emotional support should take into account and not overlook the detrimental role of academic anxiety, as it could lessen their effectiveness. Moreover, our results suggest that the improvement of emotional self-efficacy in students could play a pivotal role in counteracting school burnout. Previous studies, indeed, have shown the effectiveness of emotional intelligence-based interventions and their impact on students’ well-being (e.g., [113,114]).

The current study presents several limitations. Due to the cross-sectional design, it is not possible to draw causal relationships among the studied variables. Future longitudinal studies could help to clarify the causal direction of the observed effects and relations. Besides this, although the administrations were conducted in a single phase in the first semester of the school year, the study involved both first year and last year students. The latter may be more inclined to high anxiety than their younger peers because they have to pass the final exam, which plays a crucial role in university access.

Furthermore, the unbalanced sample does not allow us to perform further analyses to clarify the role of gender in the observed paths. Besides this, future studies could also explore reversed models that were not tested in the present study. Among the limitations, we should also mention the absence of the evaluation of the academic achievement, which could play an important role in these variables, as demonstrated by previous studies (e.g., [115,116]). Furthermore, in future studies we should analyze the relationship between the constructs studied in this work and personality traits, which can also play an important role, as shown by previous scholars (e.g., [117]). Moreover, since we did not adopt a multi-informant design, we did not collect any information on teachers. It is possible that students feel anxious because their teachers effectively do not have the necessary skills to provide emotional support. Finally, subsequent researches could verify other dynamics (i.e., mediation hypothesis) in the path trait emotional intelligence-anxiety-school burnout that was not explored in the current study.

## 5. Conclusions

The present study aimed to explore the role of trait emotional intelligence, perceived teacher emotional support, and academic anxiety on school burnout in high school students. We found that both trait emotional intelligence and perceived teacher emotional support inversely predict school burnout and that academic anxiety moderates the relation between perceived teacher emotional support and school burnout. These findings highlighted that, especially in the school context, the sole improvement of resources is not enough but there is a need to simultaneously reduce the demands. Besides this, practical interventions for burnout prevention in school settings should straighten teacher emotional support without underestimating the harmful effects of academic anxiety in students’ daily experiences and perceptions. From this perspective and according to the existing literature, applying mindfulness in schools could represent a beneficial practice. For instance, previous studies have shown the efficacy of mindfulness-based interventions in reducing anxiety and stress in students [118,119]. Furthermore, recent studies have demonstrated that mindfulness-based practices significantly improved teachers’ emotionally supportive interactions with students (e.g., [120]). Thus, planning mindfulness-based programs in high school students and training teachers in using them may contemporarily reduce the effect of adverse outcomes in students and strengthen the quality of student–teacher interactions and their perceptions of being emotionally supported.

## Figures and Tables

**Table 1:** Descriptive statistics and correlation matrix.

Variables	M	SD	Min	Max	Skewness	Kurtosis	2	3	4	5	6
1. AGE	16.27	1.48	14.00	19.00	0.26	−0.76	-	−0.02	−0.11 *	0.05	0.24 ***
2. SEX	-	-	-	-	-	-		0.11 *	0.09 *	−0.25 ***	−0.17 ***
3. TEI	4.47	0.72	1.57	6.33	−0.03	0.38			0.18 ***	−0.39 ***	−0.40 ***
4. TES	43.94	12.94	15.00	75.00	−0.07	−0.21				−0.13 **	−0.36 ***
5. AA	20.98	6.68	7.00	35.00	0.16	−0.57					0.47 ***
6. SBI	28.86	10.25	9.00	54.00	0.30	−0.38					

Note. Raw data correlation matrix. Sex: 0 = female, 1 = male. TEI= Trait emotional intelligence, TES= Teacher emotional support, AA= Academic anxiety, SBI= School Burnout, * *p* < 0.05, ** *p* < 0.01, *** *p* < 0.001.

**Table 2:** Latent moderated structural equation modeling estimations of independent variables (i.e., perceived teacher emotional support and trait emotional intelligence) and academic anxiety on school burnout.

	School Burnout	
	M0 [95% CI]	M1 [95% CI]
*Main and Interaction Effects*		
TES	−0.30 ***[−0.40, −0.22]	−0.29 ***[−0.38, −0.21]
TEI	−0.20 ***[−1.57, −0.54]	−0.19 ***[−1.52, −0.52]
AA	0.42 ***[0.52, 1.24]	0.51 *[0.19, 1.95]
TES × AA		0.13 **[0.02, 0.12]
TEI × AA		−0.01 [−0.22, 0.15]
Δ-2LL _(d.f.diff.)_ = 12.42 (2) **		

Note. CI = Confidence Interval; M0 = Model 0; M1 = Model 1; TES= Teacher emotional support, TEI= Trait emotional intelligence, AA= Academic anxiety; LL=log-likelihood; Δ-2LL=log-likelihood (LL) difference test; d.f.=degrees of freedom, * *p* < 0.05, ** *p* < 0.01, *** *p* < 0.001.
